# Relationship between computer game addiction and low mood in children

**DOI:** 10.1192/j.eurpsy.2021.1516

**Published:** 2021-08-13

**Authors:** N. Kiseleva, S. Kiselev

**Affiliations:** 1 Laboratory For Brain And Neurocognitive Development, Ural Federal University, Ekaterinburg, Russian Federation; 2 Clinical Psychology, Ural Federal University, Ekaterinburg, Russian Federation

**Keywords:** computer game addiction, low mood

## Abstract

**Introduction:**

Children with computer game addiction have a risk for development of deficit in mental functions. What kind of specific effect does this new “digital environment” have for children?

**Objectives:**

The goal of this research is to check the hypothesis that there is relationship between computer game addiction and low mood in 8-year-old children.

**Methods:**

We used questionnaire for parents to reveal children with computer game addiction. Experimental group consisted of 24 8-year-old children with computer game addiction. Control group consisted of 24 children without computer game addiction. The children from experimental and control group were matched for gender. We used Revised Children’s Anxiety and Depression Scale (RCADS) for assessment of separation anxiety disorder, social phobia, generalized anxiety disorder, panic disorder, obsessive compulsive disorder, and low mood (major depressive disorder) in children (Child Self-Reported).

**Results:**

Spearman correlation analysis has revealed the significant (p<0.05) positive correlation between level of computer game addiction and low mood in children. However, we did not find the correlation between level of computer game addiction and other scales of RCADS.

**Conclusions:**

It can be assumed that digital environment is a risk for increasing low mood in children. However, we need to do additional research using experimental design to approve the hypothesis that computer game addiction can cause the low mood in children.

